# Psychedelic researchers’ own experiences of psychedelic substances, their link to opinions of psychedelics, and reflections on positionality

**DOI:** 10.1007/s00213-025-06871-2

**Published:** 2025-08-11

**Authors:** Jussi Jylkkä, Aila Mustamo

**Affiliations:** 1 Department of Psychology, Faculty of Arts, Psychology and Theology, Åbo Akademi University, Fabriksgatan 5, Abo, 20500 Finland; 2 Folkloristics, The School of History, Culture and Arts Studies, University of Turku, Turku, Finland; 3 Cultural Anthropology, The Faculty of Humanities, University of Oulu, Oulu, Finland

**Keywords:** Psychedelics, Personal experience, Self-experimentation, Positionality, Psychedelic communities, Disclosure, Bias

## Abstract

**Rationale:**

Anecdotal evidence suggests that psychedelic researchers often have personal experiences with psychedelic substances. While such experiences may benefit research, concerns have been raised about potential biases and “excessive enthusiasm.” However, the prevalence of personal experiences, their perceived relevance, and their association with opinions about psychedelics remain underexplored.

**Objectives:**

This study aimed to investigate how common personal psychedelic experiences are among psychedelic researchers, their perceived relevance to research, and whether personal use is associated with opinions about psychedelics.

**Methods:**

Participants (*N* = 111) conducting psychedelic research in academic settings were recruited. Data were collected on personal experiences, their perceived relevance, and opinions about psychedelics. Regression analyses examined associations between personal use and opinions.

**Results:**

Most respondents (85%) reported personal experiences with classic psychedelics. On average, they saw personal experience as beneficial for research, but also as potential source of bias. They acknowledged the importance of self-reflection and the need to disclose personal experiences, but found disclosure challenging in practice. Personal use predicted more positive opinions about psychedelics’ potential to improve well-being, transform society, address the ecological crisis, and answer spiritual questions (regression βs = 0.3 – 0.5, *p*s < 0.01).

**Conclusions:**

The findings highlight the prevalence of personal psychedelic experiences among this sample of researchers and their influence on research interests and opinions. The results underscore the need for open discussion and reflection. Future studies should explore whether the observed associations reflect causal relationships or potential biases.

## Introduction

Anecdotal evidence suggests that psychedelic researchers often have personal experiences of psychedelics, and that academic psychedelic research is sometimes inspired by personal experiences (Langlitz 2012). Psychedelic researchers Peter Hendricks and Charles Nichols call the researchers’ personal experiences “the elephant in the room”, something everyone knows but no one discusses publicly (Hendricks and Nichols 2023).

Personal experience of psychedelics can be argued to be central in certain fields of research. From a philosophical perspective, it could be argued that research into the subjective effects of psychedelics requires first-hand experience, due to the so-called epistemic gap between subjective experience and science (Jylkkä 2022; Nagel 1979): knowing the nature of an experience requires having it. Indeed, some philosophers have explored the philosophical implications and interpretations of their personal psychedelic experiences (Shanon 2002; Sjöstedt-Hughes 2022). It could be argued that personal experience of a psychedelic-facilitated altered state of consciousness is necessary for understanding the psychological processes involved (Phelps 2017, p. 466). While there is lively debate regarding the importance of personal experience for psychedelic therapists (Villiger 2024), evidence suggests that majority of psychedelic therapists do have personal experiences (Aday et al. 2023).

On the other hand, it has been argued that personal experiences could threaten the objectivity of psychedelic research (Kious et al. 2023). For example, psychedelics often induce deeply meaningful or mystical-type experiences that might positively bias perceptions of their potential benefits (Ko et al. 2022). These substances can shift worldviews toward spirituality and away from scientific naturalism (Jylkkä et al. 2024; Nayak et al. 2023; Timmermann et al. 2021), sometimes leading researchers to adopt specific philosophical beliefs (Richards 2016; Smith 2000). While psychedelics can produce profound, belief-altering insights (Laukkonen et al. 2023), critics argue that psychedelics might promote false insights by reducing the influence of established beliefs and amplifying bottom-up cognitive signals (McGovern et al. 2024). Through these subjective and neurocognitive effects, psychedelics could affect or even bias the opinions of psychedelic researchers. Kious et al. (2023) describe this as “excess enthusiasm,” where positive views stem from personal psychedelic experiences rather than scientific reasoning. This raises the need to investigate whether researchers’ personal use of psychedelics correlates with more favorable opinions of their potential.

Personal use of psychedelics among psychedelic researchers can be conceptualized in terms of *positionality*, a concept often used in humanities and social sciences research to approach the relationship between a researcher’s personal background (such as experiences, opinions, cultural identifications, social class, gender identity, or nationality) and their research (Davis and Khonach 2020; Folkes 2023; Rowe 2014). Such factors could influence research in various ways, such as choice and formulation of research questions, methodologies (e.g., the theoretical approaches, choice of psychometric instruments, or interpretation of qualitative data), as well as how the results are reported (e.g., contextualization and suggested implications). In qualitative research in particular, researchers’ reflection and disclosure of their positions have been argued to be essential for methodological and ethical integrity (e.g. Folkes 2023), but it is less discussed in other fields of research (e.g. psychology, psychiatry, neuroscience).

In the case of psychedelics research, positionality includes, in addition to personal use, cultural identification. Personal use of psychedelics is often tied to various psychedelic subcultures or communities (Hartogsohn 2020) but there is no empirical research on psychedelic researchers’ identification with psychedelic subcultures. Moreover, it is not known how psychedelic researchers conceive of questions related to positionality, such as disclosure of personal background or the relevance of personal experience for research.

Irrespective of whether personal use or association with subcultures constitutes a risk of bias for research, public opinion of psychedelic research can be negatively impacted if researchers are known to personally use psychedelics or are associated with stereotypical psychedelic subcultures. Forstmann and Sagioglou (2021) found that such associations reduced perceptions of a researcher’s integrity. Similarly, the Institute for Clinical and Economic Review (ICER) criticized the clinical MDMA trials of Multidisciplinary Association for Psychedelic Studies (MAPS, later Lykos Therapeutics) for involving researchers and participants from pro-MDMA milieus (Mustafa et al. 2024), highlighting concerns about subcultural influence on evidence evaluation. This poses a challenge for researchers who wish to be transparent about their positions: while the risk of bias is debatable, it can still undermine public trust in the research. However, it remains unclear how psychedelic researchers view disclosing their positionality.

### Aims of the present study

The aims of the present study were fourfold: First, we aimed to assess the extent to which academic psychedelic researchers have experience of various psychoactive substances that can be considered as, broadly speaking, psychedelic, and whether they identify with psychedelic communities. Second, we were interested in knowing how the researchers feel regarding questions related to positionality, such as the relationship between personal experiences and research, and perceived importance of disclosure of their personal experiences or cultural identifications. Thirdly, we were interested in the researchers’ opinions about the transformative potential of psychedelics, which could be taken to reflect excess enthusiasm (Kious et al. 2023). Fourthly and finally, we were interested in whether the opinions of transformative potential are associated with personal use of psychedelics or belongingness to a psychedelic community.

## Method

### Materials

We utilized an anonymous Internet survey on the platform QuestionPro that consisted of four sections. The full survey is available at the Open Science Framework (OSF; Appendix A, https://osf.io/9qnmu/).

The first section collected background information (e.g., age, gender, education, academic expertise) and focused on participants’ academic psychedelic research, including duration, primary field (e.g., psychology, neuroscience, medicine), and methods (qualitative, quantitative, or other). No detailed demographic information such as country of residence was collected to protect anonymity. The next section assessed participants’ use of psychoactive substances, including classic psychedelics (e.g., LSD, psilocybin), analogs (e.g., 2 C-B), cannabis, empathogens (e.g., MDMA), and dissociatives (e.g., ketamine). Responses were recorded on an ordinal scale (*Never* to *Over 100 times*). Participants also estimated their average frequency of classic psychedelic use and the proportion of experiences before/after starting psychedelic research. Section three explored the relationship between personal experiences and research, including positionality and openness about use. Closed questions used a 7-point Likert scale (*strongly disagree* through *neutral* to *strongly agree*), while open questions will be analyzed separately. Finally, section four examined participants’ opinions on psychedelics’ transformative potential about treating psychiatric issues, improving wellbeing, addressing societal/ecological crises, and spiritual-philosophical questions. It also assessed views on the relevance of personal use, openness about positionality, and risks of bias, all rated on a 7-point Likert scale.

### Procedure

The participants were recruited via social media, through directly contacting psychedelic research organizations, and by directly contacting corresponding authors of recent research articles covering different aspects of psychedelics (ca. 350 persons). In the recruitment we disclosed our background as psychedelic researchers (see Appendix A at https://osf.io/9qnmu/). We aimed to target the survey to all psychedelic researchers, irrespectively of whether they have personal experience of psychedelics or not, by using recruitment texts such as “Do you study psychedelics in an academic setting? Please partake in our survey”; or “If you study psychedelics academically, please respond to our survey that probes to what extent researchers have their own psychedelic experiences”.

### Analytical approach

Regarding reflections on positionality and opinions of psychedelics, we report the mean and standard deviation, as well as proportions of participants whose response is neutral (rating = 4), agreeing (rating > 4), or disagreeing (rating < 4). Moreover, to probe whether there is a significant difference in agreeing vs. disagreeing answers, we utilized chi square tests. Neutral responses were omitted from this analysis due to their relatively smaller number, which would have artificially inflated the *p*-values. Multiple comparisons were corrected using False Discovery Rate (FDR) within two groups of comparisons: reflections of positionality (16 comparisons) and opinions of psychedelics (5 comparisons). Associations between the participant’s use of psychedelics and community identification, on the one hand, and opinions of psychedelics, on the other, were examined with multiple linear regression models. Each of the five ratings of transformative potential were included as dependent variables in separate regression models. The predictors were the use frequencies of all the psychedelic substances (classic psychedelics, psychedelic analogs, psychoactive cannabis, empathogens, and dissociatives). Identification with a psychedelic community was also included as predictor, as its possible associations with the opinions was one of our research questions, and could covary with psychedelics use. In the main regression models, no covariates were included; however, supplementary models controlling for age and gender are presented in Appendix C (https://osf.io/9qnmu/). Linear regression assumptions were inspected visually with residual plots and by examining Variance Inflation Factors (VIF).

#### Ethical approval and open data

The study was approved by the Ethics Board of the Departments of Psychology and Logopedics at the Åbo Akademi University, Finland (decision number 1/2024). The study was not preregistered. The quantitative data is available at the OSF (https://osf.io/9qnmu/), but the qualitative data will not be shared to protect participant anonymity.

## Results

### Participants

Altogether 142 participants started the survey and 116 completed the whole survey. The dropouts (*n* = 26, 18%) were excluded from further analysis. We also excluded all those (*n* = 5) who responded “No” to the question “Do you conduct psychedelic research in an academic setting?”. Thus, the final sample was *N* = 111 (29 female, 78 male, 4 other), with average age of 40.41 years (*SD* = 12.13). Majority had PhD (*n* = 66, 59%), followed by master’s degree (*n* = 31, 28%) and bachelor’s degree (*n* = 14, 13%). Regarding academic position, most (*n* = 42, 38%) reported being senior researchers, followed by PhD student (*n* = 29, 26%), master-level student (*n* = 17, 15%), postdoctoral researcher (*n* = 16, 14%), and “other” (*n* = 7, 6%). Most reported that they had conducted academic research on psychedelics for two years (*n* = 24, 22%), followed by five years (*n* = 17, 15%), and one year (*n* = 12, 11%). Most common reported primary field of research was medicine (*n* = 29, 26%), followed by psychology (*n* = 26, 23%), neuroscience (*n* = 22, 20%), philosophy (*n* = 14, 13%), chemistry/pharmacology (*n* = 8, 7%), anthropology/cultural studies/sociology (*n* = 6, 5%), and other (*n* = 6, 5%)^1^ Regarding their primary method of research, most (*n* = 69, 62%) reported using quantitative methods, followed by qualitative (*n* = 32, 29%), and “other” (*n* = 10, 9%).

### Drug use

The use of different types of psychedelic-like drugs is summarized in Table 1. Of the participants, 85% had at least one experience of classic psychedelics, 47% of psychedelic analogs, 95% of psychoactive cannabis, 71% of empathogens, and 55% of dissociatives.

**Table 1:** Psychedelic researchers’ lifetime experience of psychedelic substances. Examples were given of drugs in each category. In the category cannabis we explicitly excluded non-psychoactive products such as CBD-oil. The question formulation was “have you ever used [followed by the drug name with examples]”

	Classic psychedelics		Psychedelic analogs		Cannabis		Empathogens		Dissociatives	
	*n*	*%*	*n*	*%*	*n*	*%*	*n*	*%*	*n*	*%*
Never	17	15	59	53	6	5	32	29	50	45
Once	1	1	9	8	3	3	12	11	12	11
2–5 times	11	10	21	19	7	6	11	10	17	15
6–10 times	13	12	10	9	3	3	13	12	7	6
11–20 times	19	17	5	5	9	8	14	13	7	6
21–30 times	13	12	2	2	7	6	13	12	2	2
31–50 times	16	14	2	2	9	8	4	4	4	4
51–100 times	8	7	3	3	7	6	7	6	8	7
Over 100 times	13	12	0	0	60	54	5	5	4	4

In the case of classic psychedelics, we also asked about average use (see Table 2). The most common response was to use them few times a year (38%), followed by once a year (18%), and never (17%). Notably, 83% reported using psychedelics at least sometimes.

**Table 2:** Average use of classic psychedelics by psychedelic researchers (“if you use classic psychedelics (e.g., LSD, psilocybin, DMT, mescaline), how often do you use them on average?”)

	*n*	%
Never	19	17
Once every four years or less often	12	11
Once every three years	2	2
Once every two years	9	8
Once a year	20	18
Few times a year	42	38
Monthly	6	5
Weekly	1	1
Daily	0	0

We were also interested in whether the psychedelic experiences took place before vs. after starting doing academic research on psychedelics. Regarding the question “Please estimate how many of your experiences of classic psychedelics […] took place *before* you started doing psychedelic research”, the most common answer was “most of them” (*n* = 42, 38%), followed by “some of them” (*n* = 27, 24%), “none of them” (*n* = 23, 21%), and “all of them” (*n* = 19, 17%). That is, 79% of the participants reported having at least some experience of classic psychedelics prior to starting research. As to the question about how many of the psychedelic experiences took place *after* starting research, the most common answer was “some of them” (*n* = 57, 51%), followed by “none of them” (*n* = 31, 28%), “most of them” (*n* = 13, 12%) and “all of them” (*n* = 10, 9%). That is, based on this question, 72% of the participants had at least some experience of psychedelics after starting research.

### Reflections on positionality

The participants’ reflections on positionality (Table 3) revealed that most agreed their personal psychedelic use sparked their interest in the field (72%) and aided their research (67%) and understanding of participants’ experiences (91%). Many also felt it was essential to reflect on their relationship to the study matter (72%) and were comfortable discussing personal experiences with colleagues (67%), though opinions were split regarding openness with participants or the public. While 41% identified with a psychedelic community, 51% did not. Most agreed that personal experience is crucial for researchers in psychology/psychiatry (61%) and humanities (53%), but views were divided for medicine/neuroscience. A significant portion (40–51%) supported openness about personal use or community belonging, and a clear majority (82%) acknowledged the risk of personal use biasing research. However, there was no consensus on whether disclosing personal use would enhance a researcher’s credibility.

**Table 3:** Reflections on personal use of psychedelics. All statements were rated on a 7-point likert scale from *Strongly disagree* (1) through *Neutral* (4) to *Strongly agree* (7). Chi square tests were used to compare the frequencies of agreeing vs. disagreeing responses to probe majority opinion; neutral responses were omitted from the Chi square tests

Statement	M	SD	Agree to some extent	Neutral	Disagree to some extent	χ^2^	*p*	*p*_FDR_	
Reflections on one’s own personal experiences									
My own psychedelic experiences sparked my interest in academic psychedelic research	5.13	2.24	72%	4%	24%	26.252	2.996 × 10^−7^	1.20 × 10⁻⁶	***
My own psychedelic experiences have helped me in conducting academic research on psychedelics	4.78	2.10	67%	8%	25%	20.745	5.247 × 10^−6^	1.40 × 10⁻⁵	***
My own psychedelic experiences have helped me to understand my study participants’ experiences (*n* = 75)*	6.05	1.08	91%	7%	3%	62.229	3.058 × 10^−15^	4.89 × 10⁻¹⁴	***
I belong to a psychedelic community	3.64	1.98	41%	9%	51%	1.198	0.274	0.292	*ns*
In my field of study, it is essential for researchers to reflect on their personal relationship to the study matter	5.10	2.02	72%	8%	20%	32.98	9.309 × 10^−9^	4.96 × 10⁻⁸	***
I can be open about my personal psychedelic experiences (or lack thereof) with my colleagues	4.96	1.70	67%	11%	23%	24.253	8.450 × 10^−7^	2.70 × 10⁻⁶	***
I can be open about my personal psychedelic experiences (or lack thereof) with my study participants	3.75	1.84	25%	36%	39%	3.169	0.075	0.100	*ns*
I can be open about my personal psychedelic experiences (or lack thereof) publicly	3.73	1.95	41%	14%	46%	0.375	0.540	0.540	*ns*
**Reflections on the perceived relevance of personal experience in general**									
Within the humanities/social sciences, it is crucial for a psychedelic researcher to have their own psychedelic experience	4.30	1.85	53%	17%	30%	7.348	0.007	0.011	*
Within medicine/neuroscience, it is crucial for a psychedelic researcher to have their own psychedelic experience	4.03	1.95	49%	14%	38%	1.500	0.221	0.253	*ns*
Within psychology/psychiatry, it is crucial for a psychedelic researcher to have their own psychedelic experience	4.59	2.05	61%	9%	30%	12.129	4.965 × 10^−4^	9.93 × 10⁻⁴	***
**Openness**									
Psychedelic researchers should be open about whether they have or don’t have personal psychedelic experiences	4.60	1.71	51%	26%	23%	12.488	4.096 × 10^−4^	9.36 × 10⁻⁴	***
Psychedelic researchers should be open about whether they identify with a psychedelic community	4.36	1.54	40%	40%	21%	6.582	0.010	0.015	*
Psychedelic researchers should be open about their social background (e.g., ethnicity, worldview, social class)	4.46	1.63	42%	36%	22%	7.451	0.006	0.011	*
**Bias and credibility**									
There is a substantial risk that psychedelic researchers own psychedelic experiences bias the results of their academic research	5.19	1.47	82%	6%	12%	58.5	2.033 × 10^−14^	1.63 × 10⁻¹³	***
Psychedelic researchers’ disclosure about their own psychedelic experiences increases the credibility of their academic research	4.24	1.73	43%	25%	32%	2.036	0.154	0.190	*ns*

*For 36 participants this question was not applicable since their study did not involve participants, or they did not have personal experiences

Regarding opinions about the transformative potential of psychedelics, there was substantial agreement about the potential of psychedelics in the context of psychiatric conditions (85% agree to some extent), improving the wellbeing of healthy people (82%), positively transforming society (58%), or answering spiritual/philosophical questions (63%). By contrast, there was more disagreement (48%) than agreement (28%) that psychedelics could aid in solving the ecological crisis (Table 4).

**Table 4:** Opinions about the transformative potential of psychedelics. All statements were rated on a 7-point likert scale from *Strongly disagree* (1) through *Neutral* (4) to *Strongly agree* (7). Chi square tests were used to compare the frequencies of agreeing vs. disagreeing responses to probe majority opinion; neutral responses were omitted from the Chi square tests

Statement	M	SD	Agree to some extent	Neutral	Disagree to some extent	χ^2^	*p*	*p*_FDR_	
Psychedelics have exceptional potential in treating psychiatric conditions	5.62	1.26	85%	7%	8%	70.146	5.508 × 10^−17^	1.82 × 10⁻¹⁶	***
Psychedelics have exceptional potential for improving the psychological wellbeing of healthy people	5.52	1.31	82%	11%	7%	69.586	7.316 × 10^−17^	1.82 × 10⁻¹⁶	***
Psychedelics can transform society into a positive direction	4.63	1.38	58%	27%	15%	27.272	1.768 × 10^−7^	2.21 × 10⁻⁷	***
Psychedelics can be used to solve the ecological crisis (e.g., climate change and mass extinction)	3.53	1.62	28%	24%	48%	5.762	0.016	0.016	*
Psychedelics are extremely important in answering philosophical and spiritual/existential questions	4.96	1.50	63%	22%	15%	32.287	1.330 × 10^−8^	2.22 × 10⁻⁸	***

### Associations between classic psychedelics use and opinions about transformative potential

We used multiple regression models to examine the associations between drug use and beliefs in the transformative potential of psychedelics. The predictors included all the drug use frequencies as well as belongingness to a psychedelic community. Bivariate correlations between age, gender, community identification, the drug use variables, and the opinions are summarized in Appendix B.

Separate models were run for each of the five dependent variables, summarized in Table 5. All the models were significant (*p*_*FDR*_s < 0.001, *R*^*2*^s = 0.23 − 0.41), except for the one with the dependent variable “Psychedelics have exceptional potential in treating psychiatric conditions” (*p*_*FDR*_ = 0.158). Agreement with “Psychedelics have exceptional potential for improving the psychological wellbeing of healthy people” was predicted by use of classic psychedelics (β = 0.32, *p* =.005) and cannabis (β = 0.31, *p* =.002), as well as identification with a psychedelic community (β = 0.28, *p* <.001). The statement “Psychedelics can transform society into a positive direction” was likewise predicted by classic psychedelics use (β = 0.35, *p* =.007) and community identification (β = 0.29, *p* =.002). The opinion “Psychedelics can be used to solve the ecological crisis (e.g., climate change and mass extinction)” was predicted by use of classic psychedelics (β = 0.34, *p* <.001) and empathogen use (β = 0.34, *p* =.009), and surprisingly, negatively by use of dissociatives (β = − 0.26, *p* =.033). Finally, the statement “Psychedelics are extremely important in answering philosophical and spiritual/existential questions” was predicted by classic psychedelics use (β = 0.50, *p* <.001), and negatively by dissociatives use (β = − 0.30, *p* =.014). Of note, dissociative use was unrelated to the opinions at the bivariate level (see Appendix B), but became a negative predictor in regression models controlling for other psychedelic use.

**Table 5:** Regression models on whether the use of psychedelics and identification with a psychedelic community predict opinions of the transformative potential of psychedelics

Psychedelics have exceptional potential in treating psychiatric conditions							
Model fit	F	*R*^2^	*p*	*p*_FDR_			
	1.59	0.084	0.158	0.158			
**Estimates**	***B***	***SE***	***95% CI Lower***	***95% CI Upper***	**β**	***p***	
Classic psychedelics	0.028	0.07	−0.111	0.167	0.055	0.694	
Psychedelic analogs	−0.084	0.09	−0.262	0.095	−0.118	0.354	
Cannabis	0.052	0.06	−0.066	0.171	0.106	0.384	
Empathogens	0.069	0.071	−0.073	0.21	0.136	0.338	
Dissociatives	−0.034	0.069	−0.17	0.102	−0.067	0.619	
Community	0.111	0.064	−0.016	0.237	0.174	0.087	
**Psychedelics have exceptional potential for improving the psychological wellbeing of healthy people**							
**Model fit**	***F***	***R***^***2***^	***p***	***p***_***FDR***_			
	12.223	0.414	2.291 × 10^−10^	1.15 × 10⁻⁹			***
**Estimates**	***B***	***SE***	***95% CI Lower***	***95% CI Upper***	**β**	***p***	
Classic psychedelics	0.168	0.059	0.052	0.284	0.320	0.005	**
Psychedelic analogs	−0.127	0.075	−0.276	0.022	−0.171	0.094	
Cannabis	0.158	0.05	0.059	0.257	0.307	0.002	**
Empathogens	0.042	0.06	−0.076	0.16	0.080	0.481	
Dissociatives	−0.08	0.057	−0.193	0.034	−0.150	0.168	
Community	0.188	0.053	0.082	0.294	0.284	6.379 × 10^−4^	***
**Psychedelics can transform society into a positive direction**							
**Model fit**	***F***	***R***^***2***^	***p***	***p***_***FDR***_			
	5.274	0.233	8.822 × 10^−5^	1.10 × 10⁻⁴			***
**Estimates**	***B***	***SE***	***95% CI Lower***	***95% CI Upper***	**β**	***p***	
Classic psychedelics	0.193	0.07	0.054	0.333	0.351	0.007	**
Psychedelic analogs	−0.128	0.09	−0.307	0.051	−0.164	0.159	
Cannabis	0.018	0.06	−0.102	0.137	0.033	0.770	
Empathogens	0.011	0.072	−0.131	0.153	0.019	0.880	
Dissociatives	−0.024	0.069	−0.161	0.112	−0.044	0.725	
Community	0.200	0.064	0.073	0.327	0.287	0.002	**
**Psychedelics can be used to solve the ecological crisis (e.g.**,** climate change and mass extinction)**							
**Model fit**	***F***	***R***^***2***^	***p***	***p***_***FDR***_			
	6.563	0.275	6.689 × 10^−6^	1.11 × 10⁻⁵			***
**Estimates**	***B***	***SE***	***95% CI Lower***	***95% CI Upper***	**β**	***p***	
Classic psychedelics	0.216	0.08	0.057	0.374	0.335	0.008	**
Psychedelic analogs	−0.003	0.103	−0.207	0.2	−0.004	0.974	
Cannabis	−0.024	0.068	−0.160	0.112	−0.038	0.728	
Empathogens	0.218	0.082	0.056	0.379	0.335	0.009	**
Dissociatives	−0.17	0.079	−0.326	−0.014	−0.260	0.033	*
Community	0.118	0.073	−0.027	0.263	0.145	0.108	
**Psychedelics are extremely important in answering philosophical and spiritual/existential questions**							
**Model fit**	***F***	***R***^***2***^	***p***	***p***_***FDR***_			
	8.446	0.328	1.802 × 10^−7^	4.51 × 10⁻⁷			***
**Estimates**	***B***	***SE***	***95% CI Lower***	***95% CI Upper***	**β**	***p***	
Classic psychedelics	0.296	0.072	0.154	0.438	0.495	7.312 × 10^−5^	***
Psychedelic analogs	−0.084	0.092	−0.266	0.098	−0.099	0.364	
Cannabis	0.072	0.061	−0.049	0.193	0.123	0.241	
Empathogens	0.079	0.073	−0.066	0.223	0.130	0.282	
Dissociatives	−0.176	0.070	−0.315	−0.036	−0.289	0.014	*
Community	0.067	0.065	−0.063	0.197	0.088	0.308	

Models where age and gender were included as covariates are reported in Appendix C (https://osf.io/9qnmu/). The results did not differ from the original analysis, except for that dissociative use became a significant negative predictor of the opinion regarding improving the psychological wellbeing of healthy people (β = − 0.25, *p* =.022). Age was a negative predictor of the opinions regarding treating psychiatric problems, improving wellbeing, and positively transforming society (βs ≈ − 0.3, *p*s < 0.01).

In all the models and for all the predictors, VIFs were below 3, indicating no multicollinearity. Assumptions were checked visually with residual plots, showing no serious violations.

## Discussion

Psychedelic researchers’ personal experiences with psychedelics may be relevant to their work but could also introduce bias. However, the prevalence of such use and researchers’ views on its relevance and disclosure remain unclear. This study aimed to: (1) assess the prevalence of psychedelic use and psychedelic community identification among psychedelic researchers; (2) explore their opinions on positionality; (3) examine their beliefs in psychedelics’ transformative potential; and (4) investigate the relationship between personal use, psychedelic community identification, and opinions on psychedelics’ transformative potential.

### Prevalence of personal use

In the present sample, most psychedelic researchers had personal experience with psychedelics, with 85% having used classic psychedelics and 95% psychoactive cannabis. This aligns with previous findings that 88% of psychedelic therapists report classic psychedelic use (Aday et al. 2023) and 86% of drug researchers overall have experience of illicit drugs (Ompad et al. 2024), though only 50% of the latter had used psychedelics. This discrepancy likely stems from our focus on psychedelic researchers specifically. Of note, the usage rates in this sample far exceed those of the general population: for example, in the US the lifetime rate of psychedelics use is 13.8%, based on data from 2015 to 2018 (Simonsson et al., 2021), and in Finland lifetime rate of LSD and psychoactive mushroom use was 3.4% and 5.3%, respectively, in 2022 (Karjalainen et al. 2023). We focus on classic psychedelics in the discussion due to their potential to shift beliefs and create “excessive enthusiasm” (Kious et al. 2023).

### Reflections on positionality

A substantial majority of the participants held that their personal experiences sparked their interest in psychedelics research (72% agreement) and had used psychedelics both prior to and after starting research. This could be due to a range of reasons, such as perceptions of transformative potential through personal use, awareness of and interest in altered states of consciousness, or perceived philosophical relevance of psychedelic states (cf. Langlitz 2012). This exemplifies how a researcher’s personal background can influence which questions they start to study and consider as important (Davies 2007). It is worth considering whether the scientific investigation of the potential benefits of psychedelics would have gained momentum at all without the positive experiences of researchers themselves – as illustrated by influential figures such as William James, Aldous Huxley, and Albert Hofmann. These aspects will be elaborated in the qualitative analysis, reported in a future article.

The majority (67% agreement) held that their own experiences were helpful for conducting their research. Given that many of the participants came from medicine (including psychiatry) and psychology (49% of the respondents), they might consider that personal experience helps to understand the psychological or psychodynamic processes involved in psychedelic-assisted therapy (cf. Phelps 2017; Villiger 2024). Personal experience and its disclosure may also enhance communication and foster trust when engaging with participants in interview-based research, such as ethnographic fieldwork or qualitative psychological studies. Moreover, it can be argued that personal experience is required when studying the phenomenology and meaning of psychedelic experiences, which are often considered as ineffable and impossible to fully communicate in language (Jylkkä 2022).

It could be said that the respondents considered personal psychedelic experiences as *epistemically beneficial* on average (cf. Letheby 2021), in line with classic figures such as Huxley (1954) and James (1882), who openly discussed personal psychedelic experience in their research. Of note, it is possible that psychedelics were also considered as helpful for research through general cognitive processes such as boosting creativity (Gandy et al. 2022), or even through treating depression and thus enabling work. On the other hand, 25% of the respondents held that personal experience had *not* helped their research, which could be due to a range of reasons – for example, it could be argued that the subjective aspects are irrelevant in fields such as psychoplastogen research or pharmacokinetics. It is also possible that some researchers simply did not consider personal experience as scientifically relevant, in line with the ideal of objective science.

Most participants agreed that personal experience was “crucial” for research in psychology/psychiatry (61% agreement), aligning with the view that subjective psychological processes benefit from first-person insight. However, agreement was lower (around 50%) for humanities/social sciences and medicine/neuroscience, possibly reflecting the diverse topics and methodologies in these fields. It is important to note that these statements used the word “crucial”, which could be taken to entail that personal experience is *necessary* for conducting research on the given topic. If half of the participants considered that one cannot do valid psychedelic science without personal experience, this would be a radical statement. This raises the question: if only those with psychedelic experience are seen as legitimate psychedelic researchers, what does that mean for outsider perspectives (cf. Skille 2022; Kusow 2003)? If personal experience is considered essential, this raises questions about exclusion, gatekeeping, and whether psychedelic science is in danger of becoming a form of epistemic tribalism. On the other hand, roughly one third of the respondents did *not* consider personal experience as crucial for research, indicating diversity in the opinions.

Most participants (72%) agreed on the importance of reflecting on their personal relationship to the study matter, which is surprising given the lack of emphasis on positionality and reflexivity in fields like medicine, psychology, and neuroscience. In these domains, the prevailing ideal of objectivity often sidelines the researcher’s subjective position. It is possible that reflexivity was seen as important to avoid risk of bias stemming from personal use, given that this risk was acknowledged by 82% of the respondents. Thus, while personal experience was perceived as beneficial for research, it was also seen as a source of bias, requiring reflexivity.

It is important to note that not only presence of personal experience could constitute a bias, but also its absence. Researchers without first-hand experience may unintentionally downplay, misinterpret, or overlook aspects of psychedelic experiences that are difficult to access through third-person methods alone. For example, a researcher without personal psychedelic experience may fail to notice the potentially therapeutic or transformative nature of such experiences *as* experiences and reduce them to neurophysiological descriptions. More generally, it is arguable that psychedelic science benefits from diversity of researcher positions which, coupled with open dialogue and reflection, could enhance methodological rigor. The key issue, then, is not whether a researcher has used psychedelics, but how openly and critically they engage with their relationship to the topic.

While 51% supported openness about personal use, openness was deemed feasible mainly with colleagues (67% agreement), not publicly (41% agreement) or with study participants (25% agreement). This could suggest a tension between valuing transparency and fearing negative consequences (Ompad et al. 2024). Notably, the inability to be open publicly limits disclosure in scientific articles, where it could help readers assess researcher positions (cf. Savolainen et al. 2023). Positionality also extends beyond personal use to include broader background factors, such as identification with psychedelic communities. In this sample, 41% identified with such communities (51% did not), and participants hesitantly supported openness about psychedelic community identification (40% for vs. 21% against) and social background in general (42% for vs. 22% against). Thus, overall, there was a cautious endorsement of disclosing personal positions, but clear support of reflexivity.

It is important to distinguish between disclosure and reflexivity. While both relate to a researcher’s relationship to their topic, they serve different epistemic functions. Disclosure refers to the act of stating one’s position – such as personal experience with psychedelics or identification with a community – but this alone does not necessarily enhance the quality or credibility of research. Without critical self-examination, mechanistic disclosure may do little to mitigate bias or improve transparency, and ritualistic “positionality statements” risk becoming shallow acts of performance that satisfy normative expectations without fostering genuine epistemic accountability (Savolainen et al. 2023; Folkes 2023). At worst, they may shift the burden of critical evaluation from the researcher to the reader, who is left to interpret how, if at all, the disclosed positions influence the research. In contrast, reflexivity involves an ongoing, self-critical engagement with how one’s assumptions, beliefs, values, and commitments shape the entire research process – from question formulation to interpretation (Davies 2007). While reflexivity does not always require personal disclosure, transparent reflexivity may, in some cases, call for articulating aspects of one’s background when they are relevant to the inquiry.

These findings raise practical questions about how researchers should address positionality. While disclosure of personal background cannot be mandated and positionality statements can be criticized, the results suggest that researchers recognize the need to reflect on and discuss how their experiences influence their work. Research communities could benefit from fostering cultures that encourage open discussions and reflections on positionality, such as how their background shapes topic selection, research questions, methodologies, and interpretations.

### Opinions of transformative potential

Belief in the transformative potential of psychedelics was high. Agreement was strongest for treating psychiatric conditions and improving wellbeing, areas supported by substantial research (Aday et al. 2020; Ko et al. 2023; Wiepking et al. 2023). There was likewise significant agreement about psychedelics’ potential to positively transform society or answer philosophical-existential questions. These ideas are mainly philosophical and cannot be empirically assessed (cf. Jylkkä 2024) and as such, they may reflect personal opinion and worldview instead of evidence-based belief. By contrast, majority disagreed that psychedelics could be used to solve the ecological crisis, contrary to what some philosophers have argued (cf. Nilsson and Stålhammar 2024).

### Associations between psychedelics use and views on transformative potential

Multiple regression models were used to examine whether the use of psychedelic substances and identification with a psychedelic community predicted opinions of the transformative potential of psychedelics. The use of classic psychedelics consistently predicted more positive views across all domains with medium strength (βs = 0.3 − 0.5), except for treating psychiatric conditions. This is in line with previous research indicating that the use of psychedelics is associated with specific types of beliefs (Jylkkä et al. 2024; Nayak et al. 2023; Timmermann et al. 2021).

Surprisingly, dissociative use (e.g., ketamine) predicted more negative views on psychedelics’ potential to address the ecological crisis or answer spiritual-existential questions. This could reflect unmeasured factors, such as personality traits linked with dissociatives use (e.g., pessimism or skepticism). Alternatively, dissociatives’ phenomenological effects, which often induce detachment rather than the interconnectedness typical of classic psychedelics, might shape users’ perspectives on societal or existential issues (Muetzelfeldt et al. 2008). It is also possible that frequent dissociatives use was linked to addiction which could co-occur with depression and pessimism, leading to more negative opinions (ibid.). Further research is needed to clarify this relationship. Of note, dissociative use predicted more negative views only when controlling for other psychedelic use and was not visible on the bivariate level, indicating that the relationship is complex and likely shaped by overlapping usage patterns.

Although the present results cannot establish causality, the consistent link between classic psychedelic use and positive opinions of their transformative potential may reflect their unique pharmacological and subjective effects, such as increased suggestibility, worldview changes, or insight experiences (Kious et al. 2023). This is supported by the lack of consistent associations with other substances, suggesting these effects are specific to classic psychedelics. Alternatively, pre-existing positive beliefs about psychedelics might lead to more frequent use, driven by expectations of beneficial outcomes. Identification with a psychedelic community also predicted positive views on psychedelics’ potential to improve wellbeing and transform society, though the direction of causality – whether community affiliation shapes opinions or vice versa – remains unclear. It is also possible that personal use, beliefs, and community identification are mutually interconnected, each reinforcing and amplifying the others.

The supplementary analyses with age and gender as covariates were in line with the original results. Interestingly, age emerged as a negative predictor of the opinions about treating psychiatric conditions, improving wellbeing, and transforming society. This negative association between age and optimistic views may reflect either a cohort effect, whereby younger individuals embrace more idealistic or transformative narratives surrounding psychedelics, or an age-related increase in scientific experience or epistemic caution, leading to more critical or measured appraisals. These interpretations are not mutually exclusive and warrant further investigation.

A longitudinal or experimental study is needed to determine if the relationship between classic psychedelic use and positive opinions is causal. Importantly, even if such a causal link exists, it does not necessarily imply bias. Researchers across fields often hold strong opinions or favored hypotheses, which they can revise based on evidence; psychedelic science is no exception. Overall, it can be argued that the quality of research should be evaluated based on its methodology rather than the researcher’s personal background (cf. Savolainen et al. 2023). Importantly, dismissing a researcher’s work solely because of their background would constitute an *ad hominem* fallacy. Nonetheless, critical self-reflection on personal beliefs and positions remains a cornerstone of good methodological practice (Davies 2007).

### Limitations

The generalizability of the findings to all psychedelic researchers is uncertain due to the convenience sample and potential sampling biases. The survey’s focus on illegal psychoactive substances might have led to underrepresentation of users if participants feared consequences despite anonymity or viewed disclosing use as a risk to the field’s credibility. Conversely, users might be overrepresented if they were more motivated to report their experiences, possibly to “come out of the closet” or share their enthusiasm. Thus, it remains unclear how representative the sample is.

The sample predominantly included researchers from medicine, psychology, and neuroscience, potentially underrepresenting fields like the humanities. On the other hand, this disciplinary distribution may accurately reflect the broader population of psychedelic researchers, given the predominance of medicine, psychology, and neuroscience in published research and conference presentations within the field. It would have been interesting to examine possible differences between the fields, but subgroup analyses were not feasible in the present study due to their small sizes, and future research could focus on specific disciplines or explore differences between them (e.g., humanities vs. science).

Generalizability is also limited by the lack of demographic data in this sample. We did not gather data of the respondents’ country of residence or cultural background to protect their privacy, as this data, coupled with other information such as research field, could be used to identify the participant among the small community of psychedelic researchers. However, during data collection the survey platform enabled us to see the rough geographical location where the responses were from, and this information could not be connected with the survey responses. Based on this information we can judge that the majority of the participants were from the US and EU. A related limitation is that we did not gather information about the legal status of psychedelics in the respondent’s country, which could have affected their views on, for example, possibility of disclosure.

The online format allowed for anonymity and easy recruitment but risked unreliable responses or bots (Litman et al. 2021; Peer et al. 2022). While no attention checks were included, open answers indicated participants were attentive and genuine. The study was not preregistered, though research questions were predetermined, and open data is available for secondary analysis (https://osf.io/9qnmu/). Finally, the cross-sectional design precludes causal conclusions about the link between personal psychedelic use and opinions, requiring longitudinal or experimental research. The question of whether personal use biases research remains unresolved.

## Conclusion

It has been speculated that psychedelic researchers are personally, not just academically, interested in psychedelics. This study, among the first to quantitatively assess this question, is in line with these suspicions: psychedelic use was common in our sample of psychedelic researchers. Personal experience was perceived as beneficial for research or possibly even a requirement, although it was also recognized as a potential source of bias. Opinions of psychedelics were on average very positive and were predicted by use of classic psychedelics, but it remains unclear whether this reflects a causal effect or poses a bias risk. The researchers in our sample acknowledged the need for critical self-reflection and openness about their experiences. This aligns with qualitative and humanities research, which emphasizes the researcher’s relationship to their topic. However, many participants reported being unable to openly discuss personal experiences, especially publicly. These findings highlight the need for more open discussions about researchers’ personal experiences and their possible impact on research, including creating “safe spaces” for such dialogue without fear of negative consequences.

## Data Availability

The anonymous quantitative data is available at the Open Science Framework (https://osf.io/9qnmu/).

## Footnotes

1 Those who had chosen the category “other” were able to write their field in an open text box. Where possible, these responses were recoded into the existing categories by the authors, but six cases could not be recoded and remained in the “other” category.
